# Colonization and development of the gut microbiome in calves

**DOI:** 10.1186/s40104-023-00856-x

**Published:** 2023-04-09

**Authors:** Yufeng Du, Ya Gao, Mingyang Hu, Jinxiu Hou, Linhai Yang, Xianghuang Wang, Wenjuan Du, Jianxin Liu, Qingbiao Xu

**Affiliations:** 1 College of Animal Sciences and Technology, Huazhong Agricultural University, Wuhan, 430070 China; 2 MoE Key Laboratory of Molecular Animal Nutrition, College of Animal Sciences, Zhejiang University, Hangzhou, 310058 China

**Keywords:** Calf, Colonization, Development, Gut health, Gut microbiome

## Abstract

Colonization and development of the gut microbiome are crucial for the growth and health of calves. In this review, we summarized the colonization, beneficial nutrition, immune function of gut microbiota, function of the gut barrier, and the evolution of core microbiota in the gut of calves of different ages. Homeostasis of gut microbiome is beneficial for nutritional and immune system development of calves. Disruption of the gut microbiome leads to digestive diseases in calves, such as diarrhea and intestinal inflammation. Microbiota already exists in the gut of calf fetuses, and the colonization of microbiota continues to change dynamically under the influence of various factors, which include probiotics, diet, age, and genotype. Colonization depends on the interaction between the gut microbiota and the immune system of calves. The abundance and diversity of these commensal microbiota stabilize and play a critical role in the health of calves.

## Introduction

Microbiota in the gut is involved in the health and development of ruminants [1], and it is crucial for the development of calves [2]. The rumen microbiome of calves forms rapidly after birth and begins to establish during early life. With the development of the gastrointestinal tract (GIT), the gut microbiome of calves changes gradually, especially during weaning [3, 4]. These changes in the gut microbiome, along with nutritional and immune functions, have potential effects on host metabolism. Therefore, to optimize the health and production efficiency of ruminants, it is important to understand the factors that affect the development of the GIT. The development of the gut microbiome is mainly manipulated through probiotics, diet, age, and genotype of the calf [5–7]. Nevertheless, there is a dynamic balance among the gut microbiome, host physiology, and diet. This dynamic balance of the gut microbiome directly influences the initial acquisition, continued development, and final stability of rumen and intestinal ecosystems [8].

Application of metabolomics, metatranscriptomics, and single-cell RNA sequencing have enabled a more accurate understanding of the composition of the GIT microbiome at different stages of calf growth along with further exploration of their functional analysis through mapping in KEGG databases. These sequencing results suggested that complex microbial communities colonized the GIT of newborn calves. The early settlement of gut microbiota influenced the performance and lifelong health of animals [9–11]. There are interactions between the gut microbiome and calf growth, and these interactions are vital to the development, growth, and health of the calf. Thus, the causal relationship between the gut microbiome and calf health and the core microbiota that links host genetics and phenotypes needs to be explored. Many investigators have focused on temporal changes in the gut microbiome of newborn ruminants, particularly after weaning [12]. However, there is still a lack of understanding of the changes and development of the gut microbiome in young ruminants. This review summarizes recent developments in our knowledge of the gut microbiome of young ruminants and aims to understand the patterns of microbiome development in young ruminants and to provide novel insights to improve gut health and production of ruminants.

## Nutritional and immune function of the gut microbiome

**Fig. 1:**
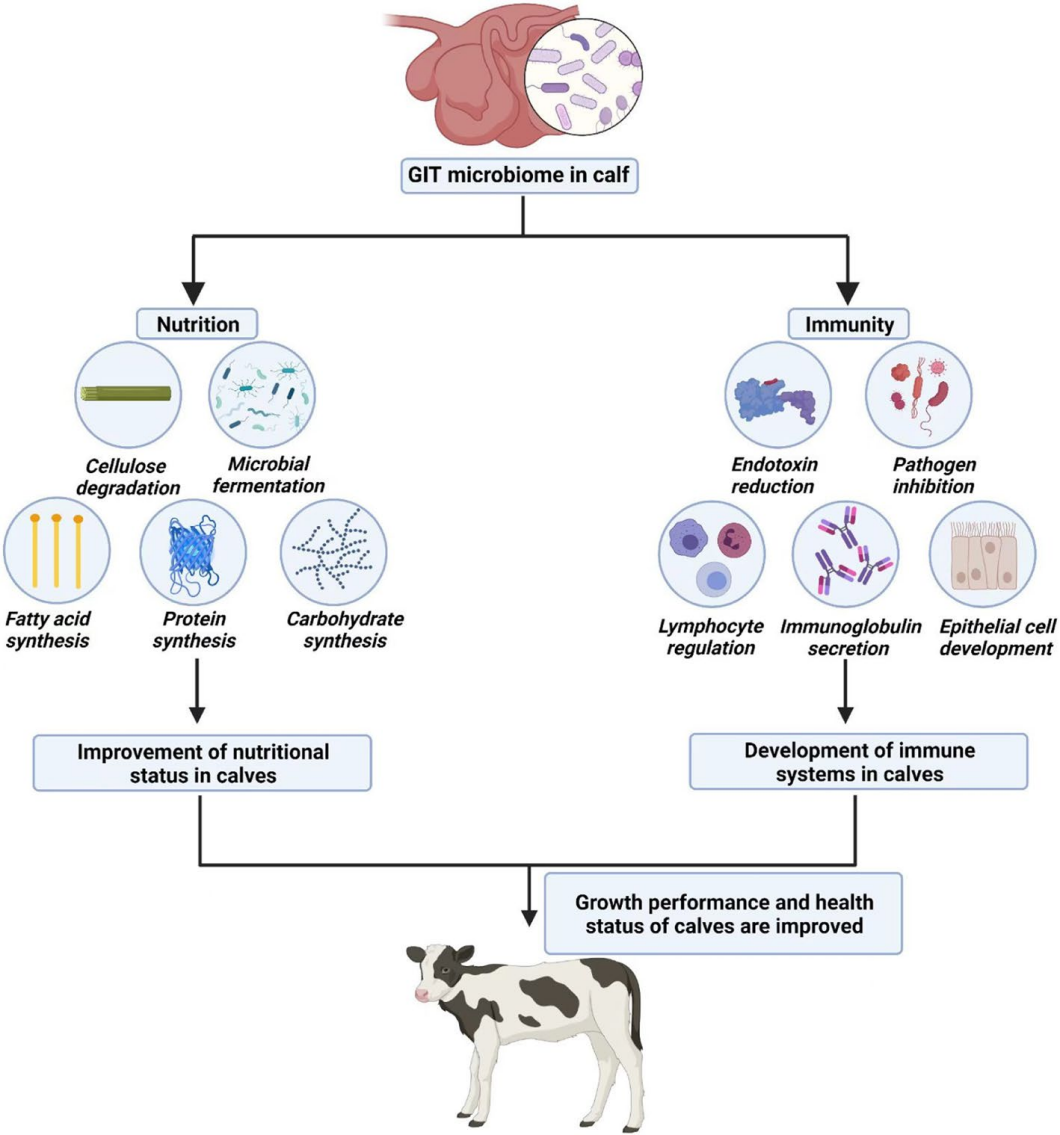
The function of the gut microbiome in calves. Created with BioRender.com

Many studies on ruminants have focused on how the microbiome affected host nutrition and immunity, which ultimately enhanced host performance and health (Fig. 1) [13–16]. Gut microbiota provides nutrition for the host through fermentation. The rumen microbiome preserves the health of its host by destroying harmful byproducts of fermentation. If the structure of the rumen microbiota is disordered, the health of ruminants is threatened. For example, when ruminant diets contained a high proportion of concentrate, lactic acid bacteria increased rapidly to produce lactic acid, which led to the death of Gram-negative bacteria; this released lipopolysaccharides and caused gastrointestinal diseases in calves [17]. Rumen microbiota was indispensable for the degradation of plant fibers [18]. Among them, hemicellulose was degraded mainly by *Prevotella* spp., whereas cellulose was degraded mainly by *Clostridium* spp. In addition, certain dominant bacteria in the rumen were related to feed efficiency [19]. In calf rumen, *Lachnospiraceae*, *Lactobacillaceae*, and *Veillonellaceae* were related negatively with high feed utilization, whereas *Methanomassiliicoccales* was related positively to feed utilization [20], suggesting that the feed efficiency may be related to specific genes of carbohydrate-active enzymes in host.

GIT microbiota influences GIT development and function by producing short-chain fatty acids (SCFAs), amino acids (AAs) or their derivatives, and bacteriocins. SCFAs were produced by the rapid fermentation of carbohydrates and were the main energy-supply substances in the epithelial cells of the GIT. Acetate coordinated interactions between epithelial and immune cells by inducing B cells to produce T-cell-dependent immunoglobulin A (IgA), which altered bacterial localization within the colon [21]. Propionate participated directly in gluconeogenesis to provide energy to calves. Supplementation with propionate induced mRNA expression of genes involved in gluconeogenesis in immortalized bovine intestinal epithelial cells [22]. In the rumen, 80% of butyrate was converted into ketone bodies, which provided > 80% of the energy for the growth of rumen epithelial cells [23]. In the intestine, butyrate provided 70% of the energy for intestinal epithelial cells through β-oxidation [24]. Butyrate increased the expression of cell cycle-related genes and decreased the expression of apoptosis-related genes, thereby regulating the proliferation of rumen epithelial cells [25]. In colonic epithelial cells, butyrate arrested the cell cycle at the G1 phase. SCFAs also regulated mitogen-activated protein kinases, sphingolipids, insulin, oxytocin, calcium, cell proliferation, and apoptosis by inhibiting histone deacetylases and activating G-protein-coupled receptors. Moreover, butyrate increased plasma GLP-2 concentration, total tract dry matter, and organic matter digestibility in lactating dairy cows [26]. Valerate increased the TEER of Caco-2 cells and reduced the paracellular permeability [27]. In rodents, valerate was correlated negatively with allobaculum, and serum valerate was potentially harmful to the health of rats [28]. In weaned piglets, with increased levels of valerate after the supplement of *Saccharomyces boulardii*, the feed conversion ratio increased and diarrhea was decreased [29]. However, no studies have focused mainly on the effects of valerate on calf development, therefore, further exploration is required.

Not all proteins were absorbed fully and utilized in the mammalian small intestine, and the remaining proteins passed through the hindgut of animals as proteins or peptides after preliminary digestion [30]. These proteins and AAs were transformed into other forms of AAs and their derivatives after fermentation by hindgut microbiota. Microbiota was involved in the metabolism of branched-chain amino acids (BCAAs). BCAAs were important for calf performance; supplementation with BCAAs (2 g/kg body weight/d; 1:1:1 of valine, leucine, and isoleucine) during nursing increased average daily weight gain in calves significantly [31]. In addition, the content of BCAAs in feces was correlated positively with calf diarrhea; however, the BCAA content in serum was correlated negatively with diarrhea, which indicated that diarrhea impaired the ability to absorb BCAAs [32].

A similar phenomenon was observed for other AAs (e.g., alanine, glycine, arginine, ornithine, and glutamic acid) [32]. The content of BCAAs and other AAs, such as plasma diamine oxidase, were biomarkers of calf diarrhea due to the negative correlation between the AA status in plasma and calf diarrhea in other studies [33, 34]. Mechanistically, angiotensin I-converting enzyme 2 was linked to AAs, microbial ecology, and intestinal inflammation by activating the mTOR signaling pathway. In the hindgut, microbiota degraded proteins and produced bioactive AA derivatives, such as tryptamine, histamine, dopamine, phenylacetylglutamine, and phenylacetylglycine [30]. In addition, tryptophan derivatives, such as indole, indoleamine-2,3-dioxygenase 1 and 2, and tryptophan-2,3-dioxygenase, were involved in the kynurenine pathway and had important implications for intestinal homeostasis [35]. Mechanistically, tryptophan derivatives acted mainly on aryl hydrocarbon receptors to participate in pro-inflammatory and tolerance responses, which meant that the remaining bacteria, such as *Limosilactobacillus reuteri* and *Lactobacillus*, exerted nutritional and immune effects on the host by altering tryptophan metabolism [36–38].

Bacteriocins are bactericidal proteins or polypeptides encoded by bacterial or archaeal genes that are synthesized by ribosomes. Bacteriocin-producing bacteria are immune to their own bacteriocins. Bacteriocins have received substantial attention because of their ability to protect the host gut against harmful bacteria [39, 40]. *Lactobacillus frumenti* prevented early weaned piglet diarrhea by secreting gassericin A, which is a class of bacteriocins [41]. In addition, when the concentration of this bacteriocin was sufficient, Lacticin 3147 prevented staphylococcal mastitis infections in cows [42].

Gut microbiota also interacts with bile acids in the host. In recent years, limited research has been conducted on the effect of bile acids on the intestinal health of calves, while the studies of bile acids have been very popular in other animals [43–46]. However, a recent study showed that gut microbial-derived ursodeoxybile acid ameliorated diarrhea effectively and improved the growth performance of calves [47]. Ursodeoxybile acid isolated from calves that were successfully treated with fecal microbiota transplantation (FMT) reduced colitis-induced *Escherichia* (*E.*) *coli* infection and hindgut microbial damage in a mouse model [47]. This study showed that the bile acids were associated with calf diarrhea, which provided pioneering insights into the development of bile acids and microbial stability in calf intestines. Overall, microbiota in the calf GIT plays a nutritional and immune role through its own metabolites, and additional, yet unknown, metabolites and their mechanisms are expected to be discovered in the future.

## Role of the microbiome on the gut barrier in calves

The protective barrier of the gut consists of mechanical, chemical, and immune components. Microbiota is necessary for the development and differentiation of the intestinal epithelium and immune system and regulates the innate and acquired immune systems in the intestine. A healthy structure of the gut microbial community is critical for host health. Under normal physiological conditions, the microbial structure in the intestine remains relatively stable, and it forms an intestinal biological barrier that inhibits the colonization of harmful bacteria and ensures calf health. Changes in gut acetate concentration also altered the responsiveness of the IgA pool to various types of bacteria [21]. IgA uncoupled and manipulated colonization during pathogenesis to promote homeostasis by neutralizing bacterial toxins or by enhancing the growth of targeted bacterial species [21, 48–51]. Intestinal bacteria, especially Gram-negative bacteria, activated intestinal dendritic cells, thereby further stimulating intestinal mucosal plasma cells to secrete IgA [52]. Moreover, intestinal commensal bacteria used their components or secretions to induce intestinal Paneth cells to synthesize antibacterial peptides through pattern recognition receptors. The resulting interactions of the microbe-related molecular pattern activated multiple signaling pathways to improve the intestinal mucosal barrier function and to promote the secretion of IgA, mucus glycoproteins, and antibacterial peptides [53], which contributed to the formation and protection of the gut barrier. For example, nisin that was obtained by calves in colostrum formed pores in the mycobacterial cell wall that reduced membrane integrity to kill *M. paratuberculosis* [54].

Intestinal commensal bacteria regulate the activity of their respective cytokines. For example, the level of IL-6 revealed the alterations in the inflammatory status of calves and cows when they suffered from GIT diseases [55, 56]. These bacteria regulated innate lymphocytes, which responded quickly to cytokine signals produced by the epithelium, such as Foxp3^+^ regulatory T cells [19, 57]. Microbial cell wall peptidoglycans maintained the structure and function of tight junctions through Toll-like receptor 2 [58]. The Gram-negative bacterium *Akkermansia muciniphila* increased the endocannabinoid content, which regulated the function of the intestinal barrier by reducing metabolic endotoxins [59]. Gut microbiota promoted the development of intestinal microvasculature by inducing angiopoietin-3 transcription factors [60]. As a result, the gut microbiota plays an important role in maintaining the structural and functional integrity of the epithelium.

The disruption of the gut barrier was accompanied by the disorder of microbial structure [61–64]. Supplementation with probiotics modulated the microbial structure through interactions with other microbiota to protect the gut barrier in newborn calves [65]. However, not all microbiota in the gut is beneficial to host health. Some genus or species of microbiota were the key biomarkers to determine the disorder of the gut microbial structure. *Clostridioides difficile* and *Clostridium perfringens*, which produced toxins, had a stonger colonization after the disorder of microbial structure [66, 67]. Key biomarkers in gut microbiota were also used to predict calf health, such as Ruminococcaceae, Lachnospiraceae, and *Phocaeicola*, *Bacteroides*, *Prevotella*, *Faecalibacterium*, and *Butyricicoccus* [68]. However, *Enterococcus*, *Ligilactobacillus*, *Lactobacilus*, *Gallibacterium streptococcus*, and *Escherichia/Shigella* were more abundant in diarrheic calves than those in healthy calves [68]. At the species level, *Eggerthella lenta*, *Bifidobacterium longum*, and *Collinsella aerofaciens* were associated with healthy status of calves, but *E. coli* and *Lactobacillus *species were associated with GIT diseases [69, 70].

Some viruses also impair the gut barrier and health of calves. Rotavirus is one of the main pathogens that cause diarrhea in calves. This infection not only reduced the richness and diversity of the intestinal microbiota significantly, but it also further disturbed the physiological homeostasis of calves [71–73]. These results suggested that the colonization of harmful microbiota interfered seriously with the stability of the host immune system, whereas beneficial microbiota improved gut health and immunity of the host.

## Dynamics of the colonization process of microbiota in calf guts

Colonization of the gut microbiota in calves is a dynamic, gradual transition from colonization to stability. The colonization of GIT microbes of calves is influenced or changed by various factors, such as dams, environment, diet, and feed supplements [74]. The process of microbiota colonization generally exhibits a certain regularity. Four processes of gut microbiota assembly have been defined in early life: dispersal, selection, drift, and diversification, which determined the priority of the infant colonization process [75]. First, high levels of aerobes and facultative anaerobes (e.g., *Lactobacillus* and *Bifidobacteria*) appeared in the GIT of the calf that consumed oxygen in the form of separate molecules, which created hypoxic conditions [76]. Subsequently, strict anaerobes (e.g., Firmicutes, Bacteroidetes, Proteobacteria, and Actinobacteria) gradually colonized and stabilized the gut.

The time at which microbiota colonized the GIT of ruminants has been a controversial research question. However, the colonization of gut microbiota was observed in ruminants at birth, and recent studies have shown that gut microbiota was already present in the gut of ruminants from the fetal period; microbiota were detected in the rumen, caecum, meconium, and amniotic fluid of calves at 5 months of gestation [77, 78]. The gut microbiota of newborn calves that were not licked by the dam was highly similar to the maternal oral microbes rather than the microbiota of the dam’s vagina, which indicated that the hindgut of newborn calves may rely on the placenta to obtain the dam’s oral or proximal GIT microbiota [79]. These studies illustrated that diverse microbiota colonized the gut before birth and changed rapidly in the early life of the calf [79–83].

**Fig. 2:**
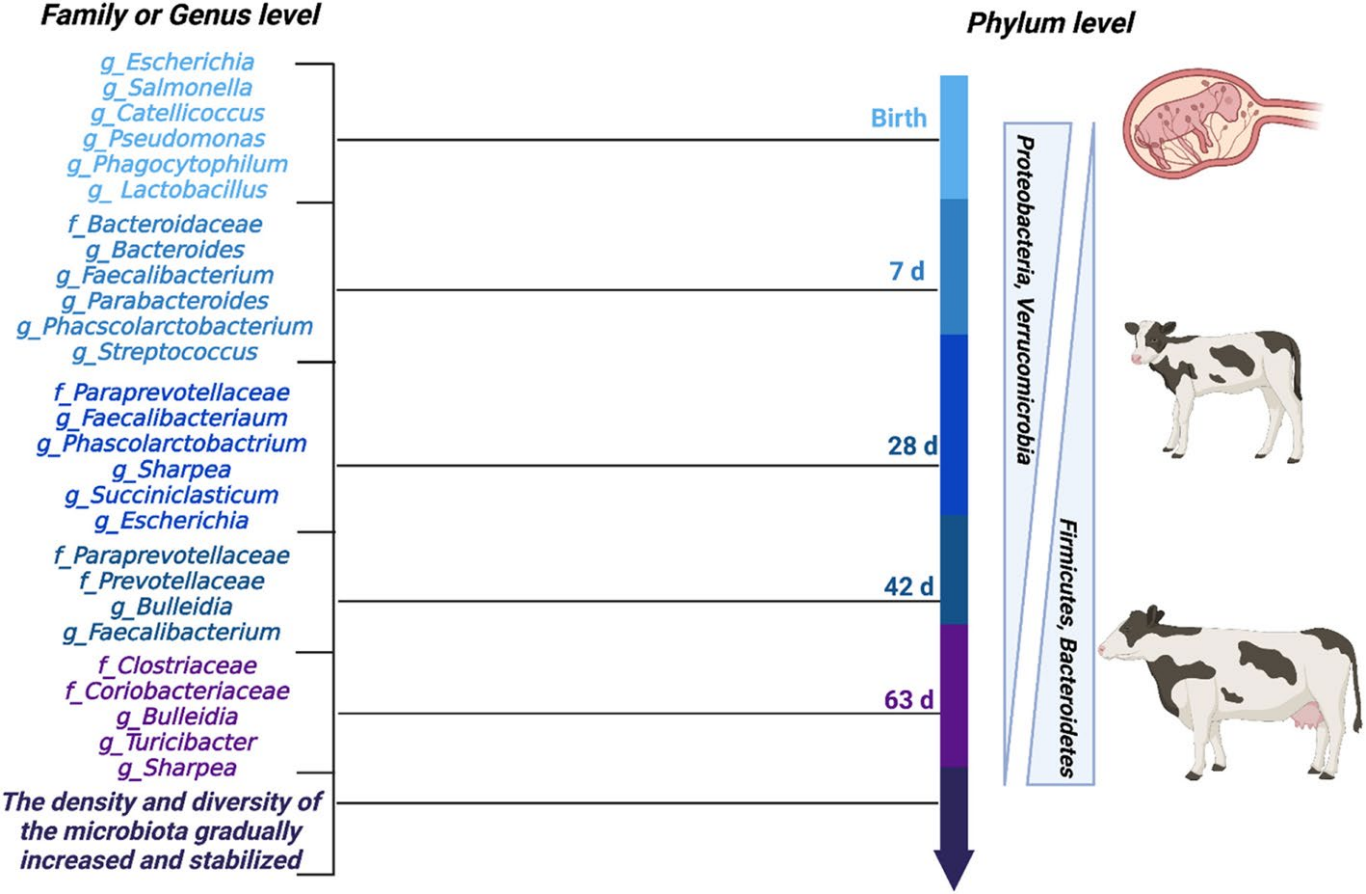
Different levels of core microbiota at different growth stages of calves. ("f_" represents family levels and "g_" represents genus levels). Adapted from previous reports [8, 12, 77, 79, 85]. Created with BioRender.com

*Escherichia*, *Salmonella*, *Catellicoccus*, *Pseudomonas*, and *Phagocytophilum* are the primary bacteria that colonized the fetuses of ruminants [84]. The intestinal bacteria of calves mainly consisted of Firmicutes, Proteobacteria, Bacteroidetes, and a small number of Actinomycetes. Numerous studies have shown that the proportion of Proteobacteria was high soon after the birth, but gradually decreased with an increase in Firmicutes and Bacteroidetes (Fig. 2) [8, 86, 87]. This may be because most Proteobacteria are facultative anaerobes, while Firmicutes and Bacteroidetes are strict anaerobes. Approximately 8 h after birth, *E. coli* and *Streptococci* colonized all GIT regions (i.e., stomach, small intestine, and cecum) of the calf, and *Lactobacillus* was detected later, and *Clostridium perfringens* was detected in the cecum; however, colonization in the other parts of the intestine was not detected at 18 h after birth [88]. Bacteria were only observed in the cecum and feces on the 2^nd^ day after birth, and in the 1^st^ week, *Lactobacillus* was dominated throughout the GIT. *Faecalibacterium* was one of the bacteria with the highest content in 1-week-old calves (21.7%); however, *Faecalibacterium* decreased with an increase in calf age. The fecal microbiota of 3-week-old calves was dominated by *Bacteroides*, *Prevotella*, *Coccus-Useriella*, and *Faebacillus* [89]. *Bacter*oides (15.3% ± 1.0%), *Prevotella* (21.6% ± 1.4%), and *Faecalibacterium* (10.3% ± 0.3%) were found in the colons of calves at the same age; however, the relative abundance of *Clostridium *XIV was only 1.6% ± 1% compared with that of the above three genera [90]. *Lactococcus flavus* and cellulolytic bacteria appeared only 5 weeks after birth; however, *Streptococcus* and *Lactococcus* were not detected [89]. *Bacteroides prevotella*, *Clostridium coccoides*, and *Eubacterium rectale* constituted the major fraction of the microbiota within 12 weeks after birth. These observations suggested that the fecal microbial community had the greatest similarity to the bacterial community in the colon; however, it did not represent the composition of the entire GIT. *Bifidobacteria* and *Lactobacilli* were able to enter the small intestine from the stomach more easily than *E. coli*, and these beneficial bacteria had a high density in the GIT of 20-week-old calves [76]. In addition, the diversity metrics (i.e., observed species, Faith’s PD, Shannon index, and evenness) were changed rapidly from 1 to 9 days of age in calves, and diarrheic status did not affect these metrics [91]. This evidence indicated that the gut microbiota changed with age.

Rumen has rich microbiota that plays a crucial role in digestion and fermentation, hence, colonization of the rumen has been the subject of most investigations on ruminant microbiota. Newborn calves are thought to be pseudo-monogastric creatures as the rumen is still in development. Gut microbes play a more important role in the early stages of life than later in the rumen. Interestingly, there are significant differences in microbial structure in different regions of the GIT of ruminants [92]. *Prevotella* and *Fibrobacter* were predominant in the stomach, while *Bacteroides*, *Clostridium*, *Alistipes*, and *Ruminococcus* were prevalent in the large intestine, and *E. coli* was high abundant in the small intestine [93]. Presumably, this difference was due to the structural characteristics and functional differences of each region of the GIT. The intestine is important for feed digestion, nutrient absorption, and the immune system of young ruminants. The main bacteria in the feces of adult cows were Firmicutes (63.7%), Proteobacteria (18.3%), Bacteroides (7.6%), and Actinomycetes (6.8%) [94].

Compared with the dominant bacteria in the rumen, species of Firmicutes were more abundant in the intestine (i.e., duodenum, jejunum, ileum, cecum, colon, and feces), while Spirochaetes and Bacteroidetes were less abundant, and Tenericutes were lower only in the large intestine (i.e., cecum, colon, and feces) [92]. At the family level, Firmicutes and Proteobacteria were more abundant in the small intestine than in other GIT compartments, and bacteria such as *Fibrobacter*, *Treponema*, and *Methanobrevibacte* were present in the rumen but not in feces [92]. However, *Methanobrevibacte* was much more abundant in the small intestine (3.7%) than in the stomach (0.71%) or large intestine (1.1%) [93]. This indicated that even if bacteria were present in the rumen, the microbiotic difference between the intestine and rumen would make bacterial colonization difficult and influence bacterial abundance.

The expression pattern of Toll-like receptor (TLR) may be one of the reasons for regional differences in gut bacteria [90, 95]. The expression of TLR1 was highest in the ileum, followed by the jejunum, and lowest in the gastric mucosa of 3-week-old calves [95]. The hindgut of young ruminants is the main site of microbial fermentation in early life. Pioneer colonization by microbiota in the hindgut may be of maternal origin, which provides the basis for a newborn calf to utilize nutrients in milk [96]. Firmicutes, Bacteroidetes, and Proteobacteria are the dominant microbial phyla in the hindgut. The relative abundances of microbial genes involved in AA metabolism, carbohydrate metabolism, and energy metabolism were enriched in these microbiota, which indicated the importance of hindgut microbiota in fermentation during the pre-weaning period [97]. As neonatal calves have an underdeveloped rumen during this period, they rely on hindgut microbial fermentation to decompose undigested dietary components. This results in the production of key metabolites such as SCFAs, AAs, and vitamins, which may be absorbed by the hindgut to promote calf growth and development.

In fact, individual differences in the hindgut microbiome affected the growth status of calves [98]. The mucosa-adherent microbiota were significantly different from the microbiota in the lumen of human and rodent guts [99–101], which was also true for ruminants [102]. The distribution of microbiota in mucosa and lumen is also different. *Bifidobacteria* and *Lactobacilli* were the most abundant microbiota that adhered to the mucous membranes in the GIT of calves (9–11 months old) and sheep (6–9 months old) [103]. At 3 weeks after birth, different bacteria have adhered to the gut mucosa of the calves [90, 104]. The bacterial abundance in the ileal mucosa was higher than that in other regions of calves [104], although most mucus-attached microbiota cannot be classified at the genus level. Above all, the dominant microbiota in different regions of the calf GIT is different at the phylum and genus levels, which is due to niche exclusion and the difference in the morphological structure of each region. Therefore, an in-depth understanding of the early colonization of gut microbes and the factors that influence the establishment of microbiota can provide a basis for reasonable control of calf gut health.

## Factors that affect the developmental process of microbiota in calf GIT

The change in the type and quantity of microbiota is a complex process, which is attributed to the interactions between the microbiome and the host, probiotics, diet, and age. A systematic study of microbial development in the GIT of young ruminants could improve feeding strategies, health, and calf production (Table 1).

**Table 1:** Factors that influence the development of gut microbiota of ruminants

Factor	Ruminant	Treatment	Result	Reference
Probiotics	Preweaning calves	Calves were inoculated through a rumen cannula with 250 mL of skim milk that contained potential probiotics isolated from the cow intestine (10^10^ CFU)	Probiotics reduced the level of carriage of *E. coli* O157:H7	[105]
	Calves at 7–56 d	*Lactobacillus acidophilus* or *Bifidobacterium pseudolongum* were administered every morning with milk replacer (3 × 10^9^ CFU)	Body weight was increased, and feed conversion and fecal score were improved	[106]
	Newborn Holstein calves	Compound probiotics that contained *Bifidobacterium thermophilum* (10^10^ CFU), *Enterococcus faecium* (10^10^ CFU), and *Lactobacillus acidophilu* (10^9^ CFU) were administered every morning with milk replacer	Increased body weight and reduced diarrhea incidence	[106]
	Newborn Holstein calves	Calves were fed milk daily with compound probiotics that contained *Lactococcus lactis*, *Pediococcus pentosaceus*, *Lactobacillus plantarum*, *Saccharomyces cerevisiae*, and *Kluyveromyces marxianus* (at the total dose of 2 × 10^10^ CFU)	Calves in the compound probiotic group had closer intestinal microbiota and lower rates of diarrhea than those in the control group	[65]
	Weaned calves at 78.3 ± 7.2 d	Calves were fed *Lactobacillus plantarum* and *Bacillus subtilis* (0.2 kg/ton feed)	Diarrhea was inhibited	[107]
	Newborn Holstein calves	*Saccharomyces cerevisiae* (2 × 10^8^ CFU), *Lactobacillus acidophilus* (2 × 10^8^ CFU), *Lactobacillus plantarum* (2 × 10^8^ CFU), *Lactobacillus rhamnosus* (2 × 10^8^ CFU), *Lactobacillus casei* (2 × 10^8^ CFU), *Bifidobacterium bifidium* (2 × 10^8^ CFU), *Pediococcus acidilactici* (2 × 10^8^ CFU), *Bacillus subtilis* (2 × 10^9^ CFU), and *Enterococcus faecium* (2 × 10^8^ CFU)	Digestion of NDF, growth preference, and rumen fermentation improved	[108]
Diet	Beef steers	Beef steers were fed with the step-up diets that contained grain and hay at ratios of 20:80, 40:60, 60:40, and 80:20	*Megasphaera elsdenii, Streptococcus bovis, Selenomonas ruminantium*, and *Prevotella bryantii* increased, while *Butyrivibrio fibrisolvens* and *Fibrobacter succinogenes* decreased in the rumen by high-concentrate diet	[109]
	Dairy cows	Cows were fed different ratios of fiber (88%, 76%, and 57.5%)	Fiber increased the population of Firmicutes (e.g., *Ruminococcus*, *Butyvibrio*, *Pseudobutyrivibrio*, *Oscillibacter*, and *Eubacterium*) and Fibrobacter	[110]
	Dairy cows	Cows were fed a low-fiber, high-PUFA (polyunsaturated fatty acid) diet or a high-fiber, low-PUFA diet for 21 d	*Prococcus* and *Filamentous bacilli* and increased *Rumenomonas*, and *Megafococcus ellieri* decreased by a high-fiber, low-PUFA diet	[111]
	Beefs	Beefs were fed different ratios of forage and concentrate (500:500 and 80:920) diets	High forage avoided dysbiosis associated with pathogenic species among Proteobacteria, and high forage increased drug-resistant bacteria and diseased related bacteria in the rumen	[112]
Age	Newborn calves	Newborn calves were fed for 14 or 42 d	Gut microbiome structure was different between the two age groups (14 and 42 d). *Oscillibacter* and *Paraprevotella* had a high richness at only 14 d, whereas *Porphyromonas* had higher richness at 42 d	[113]
	Newborn calves	From birth to 7-week-old	Chao1 index of the gut microbiome of calves increased from 0 to 7 weeks. Fusobacteria decreased gradually. *Lactobacillus* spp. and *Bifidobacterium* spp. reached a maximum of 15% at 4-week-old, and then decreased to 2%	[114]
	Newborn calves	From birth to adulthood	The rumen microbiota had significant age-related changes. Age decreased the diversity of bacteria and increased anaerobic bacteria and aerobic bacteria	[8]
Genotype	Twin and singleton calves		The twin calves had the same microbial community structure	[115]
	Herbivorous ruminants and non-ruminants		The community structure of different genotypes was different	[116]
	India cattle and buffalo		Indian cattle had a higher abundance of total anaerobic fungi and *Ruminococcus flavefaciens.* Buffalo had a higher abundance of cellulolytic bacteria *Fibrobacter succinogenes* and *Ruminococcus albus*	[117]
	Holstein and Jersey cows		Rumen microbiota was influenced by host species	[118]

### Probiotics

Probiotics have been used widely to improve rumen fermentation and to prevent pathogen colonization in calves. Administering probiotics to calves promotes the establishment of a beneficial gut microbiota, maintains microbiota stability, and inhibits pathogen growth. Moreover, these effects are important before weaning, which indicates that probiotic supplements are more effective in newborn ruminants than in mature gastrointestinal environments. The colonization of *E. coli* O157:H7 in the intestines of pre-weaned calves decreased with probiotic feeding [105]. The activity of carboxymethyl cellulase and xylanase in the rumen of North American cattle increased with the feeding of anaerobic bacteria, which improved the digestibility of crude protein and cellulose, daily weight gain, and feed efficiency [119]. Active dry yeast increased the abundance of *Vibrio* spp. in the rumen of calves during the first 28 days of life, which resulted in an elevated butyrate concentration [120]. Supplementation with yeast cultures also increased bacterial diversity in calf rumens, and this effect was especially significant in diets with high fiber levels [121]. Yeast cultures also increased the total number of bacteria, fungi, and protozoa in an artificial rumen [122], which suggested that yeast cultures promoted the multiplication of fiber-degrading bacteria in the rumen and laid the foundation to start feeding calves a fiber-based diet [123].

Commensal *E. coli* utilized epithelial-derived nitrates, which have an advantage when competing for nitrates with *Salmonella*; these bacteria invaded the niche of *Salmonella* to provide resistance to colonization [124]. This demonstrates the concept of niche pre-emption and the priority effect of the infant bacteria. As an action mechanism, probiotics can prevent the colonization of pathogens by pre-empting the space in the GIT. That is, they compete with harmful bacteria for nutrients or produce antibacterial substances, which reduced rumen acidity, improved milk yield, and reduced *E. coli* excretion in dairy cows [125–127]. Based on these theories, we can artificially intervene in and modulate the gut microbiota of newborn calves through probiotic supplementation. A negative correlation between *E. coli* and *Shigella* was found in the feces of healthy calves, which was further confirmed in vitro [128]. This suggested that *Lactobacillus* supplementation inhibited pathogen colonization to reduce calf diarrhea [129, 130].

Compound probiotics, a supplement contains multiple strains of live bacteria rather than just only one or two, promoted the gut bacterial communities of calves and made their microbial community composition more similar [65]. In addition, a high concentration of compound probiotics improved the immunity of calves by increasing the concentration of total serum proteins and immunoglobulin [131]. Compound probiotics also improved the fermentation capacity of calf rumens by producing SCFAs. The average daily weight gain of calves that were fed probiotics also increased significantly; however, with an increase in calf age, the effect of adding probiotics gradually decreased, presumably due to the preferential effect of pioneer microbiota [132]. However, there is still a lack of information regarding the effects of probiotics on the composition, metabolism, and immune function of the gut microbiota. Therefore, further exploration of probiotics is necessary to develop novel, valuable, and safe methods for improving the gut health of young ruminants.

### Diet

Weaning is the most stressful and important transitional period in the life of calves. At this time, multiple microorganisms have attached to the feed and colonized the calf GIT. The developing guts of the calves before weaning contained similar dominant phyla (Bacteroidetes, Firmicutes, and Proteobacteria). The intake of solid feed accelerated the initiation of rumen fermentation and substantially changed gut microbial components. In particular, fecal microbiota was richer and more uniform after weaning than the rumen microbiota [133]. Changes in dietary and feeding patterns before weaning had significant and lasting effects on the composition of the gastrointestinal microbiome of young ruminants [134–136]. Therefore, in terms of establishing the microbiota and cultivating the fermentation capacity of the rumen microbiome, diet management before weaning is important for young ruminants. Adding oat hay did not change the diversity of rumen microbes in calves; however, it changed the proportion of different microbial populations and affected the rumen pH indirectly [137]. Molasses beet pulp increased the concentration of VFA more than corn grain and promoted hindgut development in lactating cows; however, its application in calves requires further verification [138]. These studies have shown that changes in dietary composition greatly affected the composition of the gut microbiome in calves.

### Age

The diversity and stability of gut microbes increase gradually with increased age of a calf [70]. In the rumen, the proportions of Bacteroidetes, Firmicutes, and Proteobacteria varied greatly with calf age [113]. In feces, anaerobic species of bacteria increased with calf age, whereas aerobic species decreased gradually in the GIT of calves [8]. The expression of TLRs in the gastrointestinal tract of calves decreased significantly with age, which reduced the secretion of AMPs in the gut to facilitate colonization by various microorganisms [95]. However, when the gut microbial structure has not yet been stabilized, it facilitated invasion by pathogens.

Calf rumen microbiota showed age-related changes in different classifications and functions [113]. The abundance of Bacteroidetes in the rumen contents of calves increased from 18.1% at 2 weeks to 74.8% at 6 weeks of age, and this age-related difference was even more significant at the bacterial genus level [87]. *Prevotella* (33.1%) dominated the calf at 2 weeks of age; however, the proportion decreased significantly to 5.1% at 6 weeks of age [87]. These results suggested that the bacterial composition of the rumen in the immediate postnatal period was markedly heterogeneous; however, the composition of gastrointestinal microbes converged in similarity with maturation [90, 134, 139]. The increase in the number of anaerobes in 3-day-old calves indicated the emergence of a new anaerobic environmental niche in the early life of calves [8]. It is certain that these gastrointestinal microbiome changes and their physiological functions mature with the increase of calf age. In the future, the variation in rumen microbiota by age, the origin of these gut microbes, and the route of transfer to a newborn calf need to be studied further.

### Genotype

Genotype might also be an important factor that influenced changes in the rumen microbial community in ruminants [140, 141]. Differences in genotypes may influence the selection of certain species by affecting individual metabolic or physiological mechanisms. The effect of genotype on intestinal bacteria has recently been confirmed in pigs. The genotype significantly affected the abundance of *Erysipelotrichaceae* in the gut of pigs by regulating the concentration of N-acetyl-galactosamine, which indicated that the composition and abundance of intestinal bacteria were inherited to a certain extent [142]. In addition, the microbial community structure of twin calves under the same feeding conditions was the same according to metagenomics, and this structure was determined by the genotype of the host [115]. The diversity and community structure of intestinal anaerobic fungi in ruminants and non-ruminants were different, which indicated that different host genotypes have different microbial communities [116].

## Interactions between the gut microbiome and host immunity

A large number of microbiota in the intestine have an important impact on calf growth. The richness and activity of these gut microbiota are influenced by various factors, which include the intestinal environment, nutritional level, and health conditions. Therefore, there is an interaction exists between the gut microbiome and calf health.

The gut microbiome can maintain normal physiological activity and functions of the immune system in animals, which also affect the central nervous system. As discussed above, the dominant microbiomes in different GIT regions are significantly different, which leads to regional differences in the microbiome that may in turn affect immune function in different regions of the GIT that include the development and maturation of the mucosal immune system [140]. IgA, particularly secretory IgA (SIgA) is an important component of the intestinal mucosal immune system in animals. Most pathogenic bacteria release toxins by adhering to luminal epithelial cells, and SIgA can screen bacteria that are beneficial to the host by coating bacteria or eliminating harmful bacteria. However, owing to the special placental structure of ruminants, IgA cannot pass directly from the mother to the fetus, except through the colostrum. This means that newborn calves acquires IgA by consuming colostrum, and then IgA selects beneficial microbiota to colonize the gut, which is a host behavior for selecting for intestinal microbiota. However, when the host immune system has matured over time and produced sufficient IgA, the production of IgA and the ability to coat bacteria were regulated by bacterial metabolites and cytokines, such as acetate and TGF-β [21, 143].

In addition to screening colonizers using IgA, the host also achieves similar effects through the release of miRNAs, a group of small endogenous RNA molecules that are indispensable for regulating rumen development in newborn calves. They bind to coding RNA, block the translation process to regulate gene expression, and influence the integrity of the host immune function. miRNAs related to bacterial density were regulators of lymphoid tissue development that regulated the maturation of dendritic cells and development of immune cells [144]. Specific miRNAs exist in the calf GIT. Specifically, the expression levels of miR-l5/16, miR-29, and miR-196 were correlated positively with the 16S rRNA gene copy numbers in *Bifidobacterium* and *Lactobacillus* [144]. High concentrations of miRNAs were found in colostrum, which indicated that miRNAs also acted as signaling molecules that were transmitted from mothers to calves to stimulate intestinal epithelial cell proliferation, stem cell activity, and development of the immune system [145–147]. These results suggested that the gut microbiome mediated gene expression by regulating the expression level of miRNAs and then regulated the development of immune function in the early life of the calves.

The immune system of the calves can actively select the microbiota colonized in the intestine, and stimulation of the exogenous microbiota is essential for the development of the immune system of newborn calves. In particular, FMT is an effective treatment for diarrhea in young animals [41]. Owing to the different protocols, results may also be different. FMT increased haptoglobin and paraoxonase levels in the serum of calves; however, another study showed that FMT aggravated gastrointestinal diseases in calves [69, 148]. Therefore, screening high-quality donor feces and standardizing FMT procedures may be the key to the success of FMT. Evidence for the remission of diarrhea in calves by FMT suggested that successful FMT alleviated diarrheic symptoms by altering the composition and metabolites of microbiota, some of which were used as biomarkers to evaluate the effect of FMT, such as *Sporobacter* and *Selenomonas* in donors and *Lactobacillus* in recipients [32, 47, 149]. However, it remains unclear which microbial species play a decisive role in the remission of symptoms in calves with diarrhea and their specific mechanisms.

## Conclusions and further perspectives

The colonization of the gut microbiota (e.g., bacteria, archaea, fungi, protozoa, and viruses) in the early life of calves has attracted much attention. The gut microbiota, with nutritional and immune functions, plays a vital role in the behavior, immune system, growth, and rumen fermentation of calves. The composition, diversity, and richness of gut microbiota vary with age, species, diet, probiotics, sampling location (i.e., contents, mucous membrane, and feces), and gut segment (i.e., rumen and large and small intestines) of calves. There is an interaction between the gut microbiome (metabolites) and the calves (i.e., breed, immune system, and development). However, the causal relationship between the gut microbiome and biological health, their contribution to phenotypic variations, and the short- and long-term effects of microbial regulation remain unclear and require further study. The core gut microbiota that links host genetics (breeds) and phenotypes (e.g., methane emissions, gastrointestinal development, feed conversion rate, and milk production efficiency) also requires further exploration.

## Data Availability

Not applicable.

## Abbreviations

AA: Amino acid
BCAA: Branched-chain amino acid
FMT: Fecal microbiota transplantation
GIT: Gastrointestinal tract
IgA: Immunoglobulin A
PUFA: Polyunsaturated fatty acid
SCFAs: Short-chain fatty acids
SIgA: Secretory immunoglobulin A
TLR: Toll-like receptor
